# Variant‐specific effects of *GBA1* mutations on dopaminergic neuron proteostasis

**DOI:** 10.1111/jnc.16114

**Published:** 2024-04-20

**Authors:** G. Onal, G. Yalçın‐Çakmaklı, C. E. Özçelik, I. Boussaad, U. Ö. Ş. Şeker, Hugo J. R. Fernandes, H. Demir, R. Krüger, B. Elibol, S. Dökmeci, M. M. Salman

**Affiliations:** ^1^ Department of Physiology, Anatomy and Genetics, Kavli Institute for NanoScience Discovery University of Oxford Oxford UK; ^2^ Oxford Parkinson's Disease Centre University of Oxford Oxford UK; ^3^ Department of Neurology, Faculty of Medicine Hacettepe University Ankara Turkey; ^4^ National Nanotechnology Research Center, UNAM‐Institute of Materials Science and Nanotechnology Bilkent University Ankara Turkey; ^5^ Translational Neuroscience, Luxembourg Centre for Systems Biomedicine (LCSB) University of Luxembourg Esch‐sur‐Alzette Luxembourg; ^6^ Interdisciplinary Neuroscience Program, National Nanotechnology Research Center, UNAM‐Institute of Materials Science and Nanotechnology Bilkent University Ankara Turkey; ^7^ Department of Pediatric Gastroenterology, Hepatology and Nutrition, Faculty of Medicine Hacettepe University Ankara Turkey; ^8^ Transversal Translational Medicine Luxembourg Institute of Health (LIH) Strassen Luxembourg; ^9^ Parkinson Research Clinic Centre Hospitalier de Luxembourg (CHL) Luxembourg City Luxembourg; ^10^ Department of Medical Biology, Faculty of Medicine Hacettepe University Ankara Turkey

**Keywords:** Gaucher disease, GBA1, iPSC‐derived neurons, macroautophagy, Parkinson's disease, α‐Synuclein

## Abstract

Glucocerebrosidase 1 (*GBA1*) mutations are the most important genetic risk factors for Parkinson's disease (PD). Clinically, mild (e.g., p.N370S) and severe (e.g., p.L444P and p.D409H) *GBA1* mutations have different PD phenotypes, with differences in age at disease onset, progression, and the severity of motor and non‐motor symptoms. We hypothesize that *GBA1* mutations cause the accumulation of α‐synuclein by affecting the cross‐talk between cellular protein degradation mechanisms, leading to neurodegeneration. Accordingly, we tested whether mild and severe *GBA1* mutations differentially affect the degradation of α‐synuclein via the ubiquitin–proteasome system (UPS), chaperone‐mediated autophagy (CMA), and macroautophagy and differentially cause accumulation and/or release of α‐synuclein. Our results demonstrate that endoplasmic reticulum (ER) stress and total ubiquitination rates were significantly increased in cells with severe *GBA1* mutations. CMA was found to be defective in induced pluripotent stem cell (iPSC)‐derived dopaminergic neurons with mild *GBA1* mutations, but not in those with severe *GBA1* mutations. When examining macroautophagy, we observed reduced formation of autophagosomes in cells with the N370S and D409H *GBA1* mutations and impairments in autophagosome–lysosome fusion in cells with the L444P *GBA1* mutation. Accordingly, severe *GBA1* mutations were found to trigger the accumulation and release of oligomeric α‐synuclein in iPSC‐derived dopaminergic neurons, primarily as a result of increased ER stress and defective macroautophagy, while mild *GBA1* mutations affected CMA, which is mainly responsible for the degradation of the monomeric form of α‐synuclein. Overall, our findings provide new insight into the molecular basis of the clinical variability in PD associated with different *GBA1* mutations.

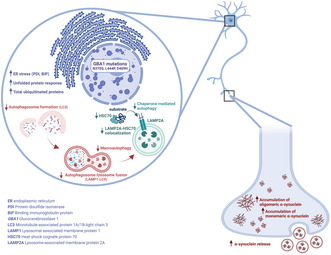

## Graphical abstract

Glucocerenrosidase1 (*GBA1*) mutations cause disruptions in endoplasmic reticulum (ER) and lysosome‐related proteostasis pathways in induced pluripotent stem cell (iPSC)‐derived dopaminergic neurons. Severe *GBA1* mutations lead to increased ER stress (increased binding immunoglobulin protein (BiP) and protein disulfide isomerase (PDI) levels), total ubiquitination rates, and unfolded protein response (UPR). Defective chaperone‐mediated autophagy (decreased heat‐shock cognate protein 70 (HSC70) levels and decreased co‐localization of lysosomal‐associated membrane protein 2A (LAMP2A) and HSC70) is observed in iPSC‐derived dopaminergic neurons with mild homozygous *GBA1* mutations. Decreased autophagosome formation (decreased microtubule‐associated protein light chain 3A (LC3) levels) is observed in iPSC‐derived dopaminergic neurons with homozygous N370S and D409H *GBA1* mutations, while fusion defects (decreased lysosomal‐associated membrane protein 1 (LAMP1) and LC3 co‐localization) are observed in neurons with homozygous L444P *GBA1* mutation. Severe *GBA1* mutations trigger the accumulation of monomeric and oligomeric forms of α‐synuclein. *GBA1* mutations increase the release of α‐synuclein from iPSC‐derived dopaminergic neurons.

**Abbreviations**
11‐MUA: 11‐mercaptoundecanoic acid
4Mu‐β‐Glc: 4‐methylumbelliferyl β‐D‐glucopyranoside
AA: ascorbic acid
ALP: autophagy–lysosome pathway
ANOVA: analysis of variance
AU: arbitrary units
Aβ: amyloid beta
BiP: binding immunoglobulin protein
CHIR: CHIR99021
CMA: chaperone‐mediated autophagy
CSF: cerebrospinal fluid
D: dissipation parameter
DM: dorsomorphin
DMEM: Dulbecco's Modified Eagle's Medium
EDC: 1‐ethyl‐3‐(3‐dimethylaminopropry) carbodiimide
ER: endoplasmic reticulum
ERAD: ER‐associated degradation
FBS: fetal bovine serum
GBA1: glucocerebrosidase 1
GCase: β‐glucocerebrosidase
GD: Gaucher's disease
HSC70: heat‐shock cognate protein 70
iPD: idiopathic PD
iPSC: induced pluripotent stem cell
LAMP1: lysosomal‐associated membrane protein 1
LAMP2A: lysosomal‐associated membrane protein 2A
LIMPII: lysosome membrane protein II
MAP1LC3A or LC3: microtubule‐associated protein light chain 3A
NHS: N‐hydroxysuccinimide
PD: Parkinson's disease
PDI: protein disulfide isomerase
QCM: quartz crystal microbalance
QCM‐D: dissipation quartz crystal microbalance
RBD: rapid eye movement sleep behavior disorder
REM: rapid eye movement
RFU: relative fluorescence unit
ROI: region of interest
SB: SB43152
smNPCs: small molecule neuronal progenitor cells
UPDRS: unified Parkinson's disease rating scale
UPR: unfolded protein response
UPS: ubiquitin–proteasome system
WES: automated western blot system
WT: wild‐type

## 1 INTRODUCTION

Mutations in the glucocerebrosidase 1 (*GBA1*) gene, encoding β‐glucocerebrosidase (GCase), are the most common genetic risk factor for Parkinson's disease (PD) and other synucleinopathies (Salman et al., 2021; Sidransky et al., 2009). GCase is a lysosomal glycoside hydrolase responsible for the degradation of lysosomal glucosylceramides and glucosylsphingosines (Liou et al., 2006). Homozygous *GBA1* mutations lead to Gaucher disease (GD), the most prevalent lysosomal storage disorder, characterized by substrate accumulation primarily in the lysosomes of macrophages because of GCase enzyme deficiency. GD is clinically classified into three subtypes based on the presence and severity of neurological symptoms: Type I (non‐neuropathic form, GD1), Type II (acute infantile neuropathic form, GD2), and Type III (chronic neuropathic form, GD3) (Brady, 1969). *GBA1* mutations can be classified as mild or severe mutations based on their association with the respective clinical subtype of GD. The most common mild *GBA1* mutation, N370S, leads to a disease phenotype with milder symptoms or even an asymptomatic course, characterized by the absence of neurological manifestations in GD1 (Baris et al., 2014). On the other hand, severe *GBA1* mutations (such as 84GG, L444P, and D409H) are associated with early onset of the disease during infancy or childhood, rapid disease progression, shorter life expectancy, and more severe neurological features such as epilepsy, impaired eye movement, and cognitive deficits (as observed in GD2 and GD3 subtypes) (Beutler et al., 2005).

It has been reported that both GD patients and carriers of *GBA1* mutations have a significantly increased risk of PD (Sidransky et al., 2009). *GBA1* mutations are estimated to increase the risk of developing PD by 20–30‐fold, and it is reported that 7%–10% of all PD patients carry heterozygous *GBA1* mutations (Nalls et al., 2013; Sidransky et al., 2009). Similar to the genotype–phenotype correlation in GD, clinical studies have highlighted substantial variations in PD symptoms between individuals with mild and severe heterozygous *GBA1* mutations (Gan‐Or et al., 2015; Thaler et al., 2018). These studies have reported that carriers of severe heterozygous *GBA1* mutations have approximately three to four times higher risk of developing PD compared to carriers of mild heterozygous *GBA1* mutations, and they experience an earlier onset of the disease by approximately 5 years (Gan‐Or et al., 2015). Furthermore, PD patients with severe heterozygous *GBA1* mutations and PD patients with homozygous or compound heterozygous *GBA1* mutations have been found to have higher unified Parkinson's disease rating scale (UPDRS) scores compared to patients with mild heterozygous *GBA1* mutations, as well as higher incidence of rapid eye movement (REM) sleep behavior disorder (RBD) and hallucinations (Thaler et al., 2018). The increased intensity of PD symptoms observed in individuals with homozygous *GBA1* mutations, compared to those with heterozygous *GBA1* mutations, is suggestive of a gene dose effect (Thaler et al., 2018). It has also been observed that PD patients with severe heterozygous *GBA1* mutations exhibit non‐motor symptoms such as depression more frequently (Thaler et al., 2018). Investigating the molecular mechanisms underlying this genotype–phenotype correlation is essential for understanding the pathophysiology of PD.

A “bidirectional pathological loop” hypothesis was previously proposed to explain the molecular relationship between mutant GCase and the accumulation of α‐synuclein, where the lysosomal accumulation of the GCase substrate, glucosylceramide, could lead to intracytoplasmic α‐synuclein accumulation. In turn, accumulated α‐synuclein could inhibit the trafficking of GCase from the endoplasmic reticulum (ER) to the lysosome, resulting in decreased activity of the enzyme (Mazzulli et al., 2011). Subsequently, various hypotheses have been proposed for the pathological link between mutant GCase and α‐synuclein accumulation, leading to two main theories. GCase gain‐of‐function theory suggests that misfolded enzyme resulting from *GBA1* mutations may cause pathological cellular perturbations leading to lysosomal dysfunction, increased ER stress, impairment of the ubiquitin–proteasome system (UPS) pathway, and impaired autophagy, all of which could trigger α‐synuclein accumulation (Fernandes et al., 2016; Sardi et al., 2015; Sidransky & Lopez, 2012). On the other hand, GCase loss‐of‐function theory proposes that deficient enzyme activity leads to the dysregulation of cellular lipid homeostasis because of substrate accumulation, which may directly influence its nucleation, intracellular trafficking, and localization and ultimately lead to GCase accumulation (Sardi et al., 2015; Sidransky & Lopez, 2012).

One of the main consequences of the pathological relationship between GCase and α‐synuclein accumulation is the functional impairment in α‐synuclein degradation through the autophagy–lysosome pathway (ALP) and the UPS, which both contribute to accumulation of α‐synuclein (Fernandes et al., 2016; Osellame et al., 2013; Schondorf et al., 2014). Recent studies have investigated differential effects of various *GBA1* mutations on α‐synuclein accumulation, providing some explanation for the variations in PD symptoms. Maor et al. reported that in SHSY5Y cells over‐expressing mutant human GCase, the presence of the severe *GBA1* (L444P) mutation leads to decreased turnover rate of GCase, increased ER stress, increased accumulation of monomeric and oligomeric forms of α‐synuclein, and increased S129 phosphorylation of α‐synuclein compared to the presence of mild (N370S) mutation. In a *D. melanogaster* model, the effect of *GBA1* L444P on α‐synuclein aggregation was larger than the effect of the N370S mutation (Maor et al., 2019). Patients carrying severe *GBA1* mutations were associated with positive family history of parkinsonism and/or dementia, an earlier onset of disease, more profound cognitive decline, a higher prevalence of RBD, and decreased cerebrospinal fluid (CSF) levels of total α‐synuclein (Lerche et al., 2020). It was also reported that the levels of phosphorylated α‐synuclein in extracellular vesicles derived from fibroblasts of PD patients with severe mutations were significantly increased compared to those with mild mutations (Cerri et al., 2021). In this study, we aimed to clarify the molecular mechanisms behind the clinical variations in PD symptoms depending on the presence of mild or severe *GBA1* mutations.

In the present study, we report that mild *GBA1* mutations associated with non‐neuronopathic GD (N370S) and severe *GBA1* mutations associated with neuronopathic GD (L444P and D409H) differentially affect the cross‐talk between cellular degradation mechanisms and α‐synuclein accumulation and can play a significant role in neuronal degeneration. Our aim was to investigate and clarify the relationship between the severity of *GBA1* mutations and the accumulation and pathologic aggregation of α‐synuclein in dopaminergic neurons. We used induced pluripotent stem cells (iPSC)‐derived dopaminergic neuronal cultures obtained from individuals representing different groups, including healthy individuals (wild‐type (WT) lines), *GBA1* homozygous patients with non‐neuronopathic Type I GD (N370S/N370S) and neuronopathic Type III GD (L444P/L444P and D409H/D409H), obligate heterozygous carriers (N370S/WT, L444P/WT, and D409H/WT), idiopathic PD patients, PD patients carrying heterozygous *GBA1* mutations (N370S/WT), and healthy individuals.

Our findings reveal that homozygous *GBA1* mutations (N370S/N370S, L444P/L444P, and D409H/D409H) led to a significant decrease in GCase protein levels (which was more significant in the presence of severe mutations) and almost complete loss of GCase enzyme activity. When we analyzed the accumulation of α‐synuclein in iPSC‐derived dopaminergic neurons, the cells with homozygous L444P and D409H *GBA1* mutations exhibited a greater accumulation of oligomeric proteins compared to those with homozygous N370S *GBA1* mutations. In addition, both idiopathic PD and PD N370S mutant lines showed increased oligomeric protein (potentially α‐synuclein) levels, and the increase was significantly higher in the PD N370S line, suggesting an additive effect of *GBA1* mutations on α‐synuclein accumulation. *GBA1* mutations also led to a significant increase in the release of α‐synuclein into the culture media in both GD and PD lines, with the homozygous D409H mutation causing the most pronounced effect. When we compared α‐synuclein release within PD lines, our results showed that the presence of *GBA1* mutation in PD N370S line amplified the release of α‐synuclein compared to the iPD line, contributing to the severity of the condition. In this study, we aimed to investigate cellular proteolysis pathways. Our results showed that iPSC‐derived dopaminergic neurons carrying homozygous L444P and D409H *GBA1* mutations exhibited increased levels of ER stress and total protein ubiquitination. We observed deficits in chaperone‐mediated autophagy (CMA) in iPSC‐derived dopaminergic neurons with homozygous N370S *GBA1* mutations but not in those with homozygous L444P and D409H *GBA1* mutations. Furthermore, examination of macroautophagy demonstrated reduced autophagosome formation in neurons with homozygous N370S and D409H *GBA1* mutations, while neurons with homozygous L444P *GBA1* mutation displayed impairments in autophagosome–lysosome fusion. Consequently, homozygous *GBA1* mutations led to the accumulation and release of oligomeric α‐synuclein in iPSC‐derived dopaminergic neurons because of increased ER stress and impaired macroautophagy. Mild homozygous *GBA1* mutations affected the chaperone‐mediated autophagy, primarily responsible for degrading the monomeric form of α‐synuclein. Taken together, these findings provide insights into the molecular basis underlying the clinical differences observed in PD patients with mild and severe *GBA1* mutations.

## 2 MATERIALS AND METHODS

### 2.1 Establishing human dermal primary fibroblast cultures

The study was approved in advance by the Non‐Invasive Clinical Research Ethics Committee of Hacettepe University (approval reference number GO‐17/673). The study was not pre‐registered. Informed consent forms were obtained from each patient or their parents. The demographic information of the individuals is summarized in Table S1. We used induced pluripotent stem cells (iPSC)‐derived dopaminergic neuronal cultures obtained from 10 individuals representing different groups, including healthy individuals (wild‐type (WT) lines), *GBA1* homozygous patients with non‐neuronopathic Type I GD (N370S/N370S) and neuronopathic Type III GD (L444P/L444P and D409H/D409H), obligate heterozygous carriers (N370S/WT, L444P/WT, and D409H/WT), idiopathic PD patients, PD patients carrying heterozygous *GBA1* mutations (N370S/WT), and healthy individuals. No exclusion criteria were pre‐determined. No sample size calculation was performed. No blinding was performed.

Skin biopsy samples from the individuals were obtained using 4 mm circular Visipunch tool and kept in DMEM (Dulbecco's Modified Eagle's Medium) containing 20% FBS (fetal bovine serum), 5% penicillin/streptomycin, and 5% amphotericin B. The biopsy samples were transferred to petri dishes and washed once with PBS containing 5% penicillin/streptomycin and 5% amphotericin B, followed by two washes with PBS without antibiotic/antimycotics. The biopsy samples were then dissected into small pieces using a sterile scalpel to ensure adherence of the fragments to the petri dishes. Tissues were kept in DMEM with 3.7 g/L NaHCO_3_, 4.5 g/L D‐glucose, and stable L‐glutamine (Biochrom) medium containing 20% FBS (Biochrom), 100 mg/mL each penicillin/streptomycin (Biochrom), and 1% amphotericin B and incubated at 37°C in a humidified atmosphere with 5% CO_2_. Fibroblasts were expanded up to the 5th passage and used for reprogramming into iPSCs. Cells were routinely tested for mycoplasma contamination and found negative.

### 2.2 Reprogramming fibroblasts into iPSCs

Human fibroblasts were plated at a density of 2 × 10^4^ cells/cm^2^ and transfected with 1 μg/10^5^ cells of each pCXLE‐hUL (Addgene #27080 (RRID:Addgene_27 080)), pCXLE‐hSK (Addgene #27078 (RRID:Addgene_27 078)), and pCXLE‐hOCT3/4 (Addgene #27076 (RRID:Addgene_27 076)) plasmids using the Lonza Human Dermal Fibroblast Nucleofector™ Kit and Amaxa Nucleofector B16 program. The cells were plated on feeder‐free Matrigel‐coated (Corning BD Matrigel hESC‐qualified Matrix) plates. E8 medium (DMEM F‐12 with HEPES (Life Technologies)) supplemented with 1% penicillin/streptomycin (Life Technologies), 1% insulin–transferrin–selenium (Life Technologies), 2 ng/mL TGFβ1 (PeproTech), 10 ng/mL FGF2 (PeproTech), 64 μg/mL ascorbic acid (Sigma‐Aldrich), 100 ng/mL heparin (Sigma‐Aldrich), 10% mTesR (StemCell Technologies), and 100 μM sodium butyrate (Sigma‐Aldrich) was used and changed daily. Once colonies formed, they were isolated, replated on Matrigel‐coated plates, and split using 0.5 mM EDTA (Life Technologies) when they reached the desired confluency. The cells were incubated at 37°C in a humidified atmosphere with 5% CO_2_.

### 2.3 Differentiation of iPSCs into smNPCs and mature dopaminergic neurons

The differentiation of iPSCs into dopaminergic neurons was performed in two steps following the protocol described by Reinhardt and colleagues (Reinhardt et al., 2013) (Figure S1). In the first step, differentiation of iPSCs into small molecule neuronal progenitor cells (smNPCs) was performed. During the differentiation process, 10 μM SB43152 (SB) (Sigma‐Aldrich), 1 μM dorsomorphin (DM) (Sigma‐Aldrich), 3 μM CHIR99021 (Axon), and 0.5 μM purmorphamine (Sigma‐Aldrich) were added to the culture for 2 days. Then, the media was changed to N2B27 neuronal cell culture medium composed of 49% Neurobasal medium (Life Technologies), 49% DMEM/F12 (Life Technologies), 1% Glutamax (Life Technologies), 1% penicillin–streptomycin (Capricorn), 1:100 B‐27 supplement (Thermo Fisher Scientific), and 1:200 N‐2 supplement (Thermo Fisher Scientific), with the addition of 150 μM ascorbic acid (Sigma‐Aldrich). To ensure the homogeneity of the cell culture, smNPCs were passaged up to a minimum of 13 passages. In the second step, for the differentiation of smNPCs into midbrain dopaminergic neurons, smNPCs (>p13) were cultured in N2B27 neuronal media supplemented with 200 μM ascorbic acid (Sigma‐Aldrich), 500 μM db‐cAMP (Sigma‐Aldrich), 20 ng/mL BDNF (PeproTech), 10 ng/mL GDNF (PeproTech), and 1 ng/mL TGF‐β‐III (PeproTech) for at least 21 days as it has been reported that mature dopaminergic neuron cultures are obtained on the 21st day of differentiation using this protocol (Reinhardt et al., 2013). The concentrations of the compounds are indicated in Figure S1.

### 2.4 Immunocytochemistry

Cells were seeded on Matrigel‐coated glass coverslips placed in 24‐well culture plates. The cells were fixed with 4% paraformaldehyde in PBS for 15 min at room temperature and washed twice with PBS. Then, cells were blocked and permeabilized using 10% FBS and 0.1% Triton X‐100 (Sigma‐Aldrich) in PBS for 60 min at room temperature. The cells were treated with primary antibodies in 1% goat serum (Sigma‐Aldrich) in PBS overnight at 4°C. Following washing with PBS, the cells were incubated with secondary antibodies in 1% goat serum (Sigma‐Aldrich) in PBS for 1 h at room temperature. Cells were washed three times with PBS and incubated with DAPI for nuclear counter‐staining. The coverslips were mounted on the slides using ProLong™ gold anti‐fade reagent (Invitrogen, Thermo Fisher). Images were acquired using a Leica SP8 laser scanning confocal microscope with an oil immersion objective (63x, NA: 1.40). Fluorescence intensity and co‐localization analysis were conducted using ImageJ software. Intensity analysis involved defining the entire image as a region of interest (ROI) with the measurement of intensity expressed as the mean gray value. In order to analyze co‐localization, we used ImageJ JACoP plugin. The co‐localization was analyzed based on intensity and quantified using Pearson's *R* value and Spearman's rank correlation coefficients as a measure of linear correlation.

The primary antibodies used for immunocytochemistry were rabbit anti‐OCT3/4 (1:250) (sc5279, Santa‐Cruz (RRID:AB_628051)), rabbit anti‐NANOG (1:250) (ab21624, Abcam (RRID:AB_446437)), goat anti‐SOX2 (1:250) (sc17320, Santa‐Cruz (RRID:AB_2286684)), rabbit anti‐amyloid oligomer (1:100) (AB9234, Sigma‐Aldrich (RRID:AB_11214948)), mouse anti‐HSC70 (1:250) (sc7298, Santa Cruz (RRID:AB_627761)), mouse anti‐TH (1:200) (ab75875, Abcam (RRID:AB_1310786)), mouse anti‐Tubulin βIII (1:5000) (05559, Sigma‐Aldrich (RRID:AB_309804)), rabbit anti‐LAMP2A (1:200) (ab18528, Abcam (RRID:AB_775981)), mouse anti‐LAMP1 (1:200) (ab25630, Abcam (RRID:AB_470708)), rabbit anti‐LC3 (1:200) (L7543, Sigma‐Aldrich (RRID:AB_796155)), mouse anti‐SQSTM1 (1:200) (H00008878‐M01J, Abnova (RRID:AB_548364)), rabbit anti‐NESTIN (1:250) (ab105389, Abcam (RRID:AB_10859398)), mouse anti‐MUSASHI (1:250) (MABE268, Sigma‐Aldrich (RRID:AB_2576205)), and mouse anti‐SOX1 (1:250) (BDB560749, Fisher Scientific (RRID:AB_1727572)). Suitable Alexa Fluor‐conjugated secondary antibodies (1:500) (Invitrogen) were used against primary antibodies.

### 2.5 Immunoblotting

Cells were collected in RIPA buffer (Sigma‐Aldrich) containing a 1X cOmplete™ protease inhibitor cocktail (Roche). The samples were sonicated six times at 50% amplitude for 20 s and then centrifuged at 14 000 *g* for 20 min at 4°C. The supernatants were transferred to new tubes. The quantification of the protein samples was performed using the BCA Protein Assay Kit (Thermo Fisher Scientific) following the manufacturer's protocol. The protein concentrations were measured at 562 nm using a SpectraMax M2 spectrophotometer. For the subsequent steps, 30 μg of protein extract was mixed with 4X Laemmli buffer and denatured at 100°C for 5 min. The denatured proteins were loaded onto 12% or 15% SDS‐PAGE gels along with protein molecular weight markers. Electrophoresis was performed at 100 V for 2 h. Wet transfer protocol was applied at 100 V for 1.5 h at room temperature. The membranes were blocked with 5% non‐fat milk powder (Sigma‐Aldrich) in 0.1% TBS‐T buffer for 1 h at room temperature. Primary antibodies were diluted in 5% non‐fat milk powder in 0.1% TBS‐T buffer and incubated with the membranes overnight at 4°C. Afterward, the membranes were washed three times with 0.1% TBS‐T and then incubated with HRP‐conjugated secondary antibodies diluted in 5% non‐fat milk powder in 0.1% TBS‐T buffer for 1 h at room temperature. Following three additional washes with 0.1% TBS‐T, the membranes were treated with SuperSignal™ West Femto Maximum Sensitivity Substrate (Thermo Fisher Scientific) according to the manufacturer's protocol. Chemiluminescent imaging was performed using the GeneGnome 5 system (Syngene).

The primary antibodies used for immunoblotting are mouse anti‐LIMPII (1:1000) (ab176317, Abcam (RRID:AB_2620169)), mouse anti‐β glucocerebrosidase (1:1000) (sc166407, Santa Cruz (RRID:AB_2109068)), mouse anti‐TH (1:1000) (ab75875, Abcam (RRID:AB_1310786)), rabbit anti‐LAMP2A (1:1000) (ab18528, Abcam (RRID:AB_775981)), mouse anti‐LC3 (1:1000) (L7543, Sigma‐Aldrich (RRID:AB_796155)), and mouse anti‐GAPDH (1:1000) (G8795, Sigma‐Aldrich (RRID:AB_1078991)). Suitable HRP‐conjugated secondary antibodies (1:5000) (Invitrogen) were used against primary antibodies.

### 2.6 WES (automated western blot system)

The standard protocol of the ProteinSimple WES (Bio‐techne) automated system was followed. The protein samples at a final concentration of 0.8 mg/mL, primary antibodies, secondary antibodies, protein ladder, and washing solutions were loaded onto the assay plate as per the manufacturer's instructions. The WES Capillary Cartridge and the assay plate were inserted into the instrument. The protocol was applied at 375 V for 43 min. The results were automatically processed using Compass for Simple Western™ (Bio‐techne).

The primary antibodies used for WES are mouse anti‐α‐synuclein (1:10) (610 787, BD Biosciences (RRID:AB_398108)), mouse anti‐HSC70 (1:100) (sc7298, Santa Cruz (RRID:AB_627761)), rabbit anti‐BiP (3177, Cell Signaling (RRID:AB_2119845)), rabbit anti‐PDI (1:100) (3501, Cell Signaling (RRID:AB_2156433)), mouse anti‐Ubiquitin (1:125) (sc8017, Santa Cruz (RRID:AB_628423)), mouse anti‐SQSTM (1:50) (H00008878‐M01J, Abnova (RRID:AB_548364)), mouse anti‐GAPDH (1:1000) (G8795, Sigma‐Aldrich (RRID:AB_1078991)), and mouse anti‐β‐Actin (1:1000) (M01263, Boster (RRID:AB_3082544)). Suitable HRP‐conjugated secondary antibodies (1:5000) (Invitrogen) were used for primary antibodies.

### 2.7 4‐MUG GCase activity assay

iPSC‐derived dopaminergic neurons were seeded at a density of 5000 cells per well onto Matrigel‐coated black 96‐well plates. The cells were incubated with 2.5 mM 4‐methylumbelliferyl β‐D‐glucopyranoside (4Mu‐β‐Glc) (Sigma‐Aldrich) substrate in 0.2 M sodium acetate buffer (pH 4) at 37°C for 6 h. The reaction was stopped by adding 0.2 M glycine buffer (pH 10.8). The fluorescence emission was measured at A355/A460 (excitation/emission) wavelengths for 0.1 s using SpectraMax fluorescence plate reader. The results were normalized to the protein concentration values measured using the BCA Protein Assay Kit (Thermo Fisher Scientific) for cell lysates.

### 2.8 Quartz crystal microbalance (QCM‐D) analysis

The quartz crystal microbalance (QCM) sensor is a technique used in various bio‐detection applications to detect a wide range of bioanalytes with nanogram‐level sensitivity. It operates by measuring the change in total mass on the gold‐coated quartz crystal surface using resonance frequency (Kosslinger et al., 1992). The dissipation quartz crystal microbalance (QCM‐D) monitoring allows real‐time, label‐free measurements of molecular adsorption and/or interaction on the crystal surface. It also enables monitoring of the viscoelastic properties of the adsorbed layer using the dissipation parameter (D) by Q‐Sense. Dissipation occurs when the voltage on the crystal is cut off, resulting in the dissipation of the oscillating crystal's energy.

⍺‐synuclein expression and purification using immobilized metal affinity chromatography was performed as previously described (Ozcelik et al., 2023). Briefly, ⍺‐synuclein was expressed as a fusion to the TEVp_Recognition_Site‐GST‐6His in the E. coli BL21 (DE3) strain and then the cells were harvested and lysed. After centrifugation at 15 000 *g* for 30 min at 4°C, the supernatant was collected and filtered with 0.45‐μm cellulose syringe filters. The filtered supernatant was loaded onto a HisTrap nickel column (GE Life Sciences 17 524 701), and purification was performed using FPLC (ÄKTA start protein purification system) according to the manufacturer's specified protocol. In order to obtain the monomeric form of ⍺‐synuclein, the purified ⍺‐syn‐TEVp_Recognition_Site‐GST‐6His protein sample was digested using TEV protease. The subsequent purification of the monomeric ⍺‐synuclein from remaining proteins was carried out using the HisTrap nickel column (GE Life Sciences 17 524 701) in conjunction with FPLC (ÄKTA start protein purification system). The resulting purified monomeric ⍺‐synuclein was transferred to 1X PBS to continue the QCM analysis.

For QCM analysis, the QCM gold sensor (Biolin Scientific QSense QSX 301 Gold) was chosen. Before each measurement, the chips underwent cleaning with piranha solution (H_2_O_2_:H_2_SO_4_ (1:3)) by immersing the chip in the solution for 30 min at 80°C. To eliminate any residual solution, the chips were immersed in ddH2O for 5 min twice. Subsequently, the cleaned chips were coated with 20 mM 11‐mercaptoundecanoic acid (11‐MUA) and activated through 1‐ethyl‐3‐(3‐dimethylaminopropry) carbodiimide (EDC)/N‐hydroxysuccinimide (NHS) coupling reaction, followed by protein immobilization, as previously described (Ahan et al., 2022). A total of 500 μg of monomeric ⍺‐synuclein was immobilized onto the gold chip, and surface deactivation was performed using 1 M ethanolamine HCl. Afterward, the chip was treated with equal volumes of cell culture medium of iPSC‐derived dopaminergic neurons at Day 90 to assess the interaction with monomeric ⍺‐synuclein on the surface of QCM chip. Following each sample addition, a 1X PBS wash was conducted to remove unbound components. The amount of protein from the supernatant bound to the chip surface was quantified by measuring the first, third, fifth, seventh, and ninth overtones of frequency change. The QCM plots were generated using the average of third, fifth, seventh, and ninth frequency change values plotted against time (min). The data were normalized to the amount of total protein in each sample.

### 2.9 Data analysis

The data were evaluated using the D'Agostino–Pearson normality test. For the data with normal distribution, two‐way analysis of variance (ANOVA) and post‐hoc Bonferroni test were used. For data that did not follow a normal distribution, Kruskal–Wallis test and Dunn post‐hoc test were employed. No test for outliers was conducted. GraphPad Prism version 6.0.3 was used for statistical analysis.

## 3 RESULTS

### 3.1 Determination of β‐glucocerebrosidase (GCase) expression, activity, and trafficking in iPSC‐derived dopaminergic neurons

**FIGURE 1:**
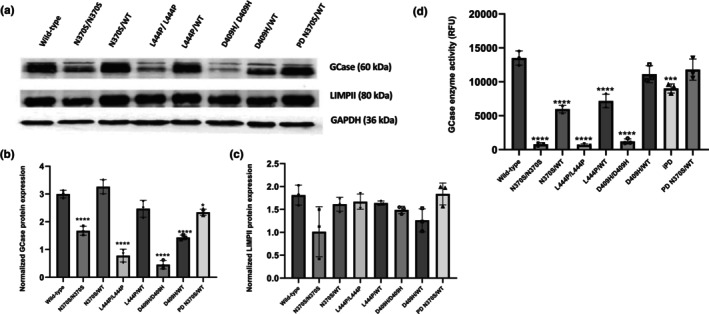
The impact of *GBA1* mutations on GCase expression, activity, and trafficking in differentiated dopaminergic neurons. (a) Western blot image showing the protein expression levels of GCase and LIMPII in induced pluripotent stem cell (iPSC)‐derived dopaminergic neurons (Day 40). Graphs showing (b) GCase and (c) LIMPII protein expression levels normalized to GAPDH expression in wild‐type and *GBA1* mutant dopaminergic neurons. Data are shown as mean + standard deviation. Ordinary one‐way ANOVA, *F*(7, 16) = 82.41, *p* < 0.0001 and *F*(7, 16) = 3.594, *p* < 0.0162, respectively. (d) GCase enzyme activity assay results in wild‐type and *GBA1* mutant iPSC‐derived dopaminergic neurons (Day 40). Data are shown as mean + standard deviation. Ordinary one‐way ANOVA, *F*(8, 18) = 94.68, *p* < 0.0001 (*n* = 3 from three different differentiations of iPSC‐derived dopaminergic neurons) (RFU, relative fluorescence unit; *p < 0.05, ***p < 0.001, and ****p < 0.0001).

We started examining the effects of *GBA1* mutations on GCase expression and intracellular trafficking in dopaminergic neurons differentiated from small molecule neuronal precursor cells (smNPCs) for 40 days as previously described (Reinhardt et al., 2013). Our results revealed a significant decrease in total GCase protein levels in N370S, L444P, and D409H homozygous *GBA1* mutant lines (Figure 1a,b). The decrease was approximately twofold for the N370S homozygous *GBA1* mutant line and threefold for L444P and D409H homozygous *GBA1* mutant lines compared to the control lines. GCase protein levels were also significantly reduced in in the PD N370S/WT line compared to the wild‐type lines. Additionally, within the heterozygous lines, the D409H/WT heterozygous line was the only line in which GCase protein levels significantly decreased compared to the wild‐type lines.

Lysosome membrane protein II (LIMPII), encoded by the SCARB2 gene, is responsible for the trafficking of GCase from the ER through the Golgi to the lysosomes especially in neuron‐like cells. To understand the possible effects of GCase mutations on the trafficking of the enzyme to the lysosomes, the expression levels of LIMPII protein were analyzed. The levels of LIMPII protein were not significantly different between *GBA1* mutant and wild‐type lines (Figure 1a,c). As we did not see any significant difference in LIMPII levels, our results suggest that LIMPII‐mediated trafficking of GCase to the lysosomes was not affected in *GBA1* mutant dopaminergic neurons.

We found that N370S, L444P, and D409H homozygous *GBA1* mutations caused almost complete inhibition of GCase enzyme activity (Figure 1d). N370S/WT and L444P/WT heterozygous *GBA1* mutant lines showed a 50% decrease in GCase enzyme activity (*p* < 0.001). No significant difference was detected in the neurons with the D409H/WT heterozygous line compared with the wild‐type lines. We also observed a significant decrease in GCase enzyme activity in an idiopathic PD patient line (iPD) and in PD N370S/WT dopaminergic neurons.

### 3.2 Accumulation of α‐synuclein in *GBA1* mutant iPSC‐derived dopaminergic neurons

**FIGURE 2:**
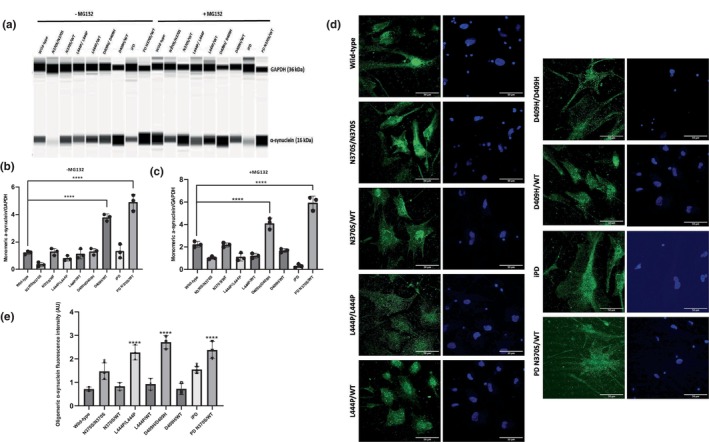
α‐synuclein accumulation and oligomerization in induced pluripotent stem cell (iPSC)‐derived dopaminergic neurons with *GBA1* mutations. (a) Immunoblot by automated western blot system (WES) analysis of intracellular monomeric α‐synuclein protein expression in iPSC‐derived dopaminergic neurons (Day40) under basal conditions and after 24‐h treatment with 100 nM MG132 proteasome inhibitor. (b) Graph depicting the optical densities of monomeric α‐synuclein bands normalized to GAPDH expression in wild‐type and *GBA1* mutant dopaminergic neurons under basal conditions and (c) upon MG132 treatment. Data are shown as mean + standard deviation. Two‐way ANOVA, (b) *F*(8, 18) = 62.45, *p* < 0.0001 and (c) *F*(8, 18) = 86.15, *p* < 0.0001 (*n* = 3 from three different differentiations of iPSC‐derived dopaminergic neurons). (d) Immunofluorescence images of oligomeric (A11) α‐synuclein (green) and nuclei (blue) in differentiated iPSC‐derived dopamine neuron cultures (Day40). (e) Quantitative analysis of immunofluorescence intensity of oligomeric α‐synuclein in dopaminergic neurons, presented as arbitrary units (AU). Data are shown as mean + standard deviation. Ordinary one‐way ANOVA, *F*(8, 18) = 26.86, *p* < 0.0001 (*n* = 3 from three different differentiations of iPSC‐derived dopaminergic neurons and at least 20 cells per condition). Significantly different groups are indicated by * and **** (*p < 0.05 and ****p < 0.0001), scale bar: 50 μm.

Next, we examined the accumulation of the monomeric form of α‐synuclein in iPSC‐derived dopaminergic neurons under basal conditions and MG132 proteasome inhibitor‐treated conditions. Upon MG132 treatment, we aimed to examine the effects of *GBA1* mutations on the accumulation of the monomeric form of α‐synuclein upon proteasome inhibition. Under basal conditions, we found a significant increase in monomeric α‐synuclein levels in PD N370S/WT line compared to the wild‐type cells (Figure 2a,b). We also found a significant increase in monomeric α‐synuclein levels in D409H heterozygous *GBA1* mutant neurons and no significant differences observed in the other groups (Figure 2a,b). Following on MG132 treatment, a significant increase in monomeric α‐synuclein levels was observed in D409H/D409H *GBA1* mutant dopaminergic neurons compared to wild‐type cells (Figure 2a,c). These data suggest that inhibition of the UPS might have a significant impact on the D409H/D409H *GBA1* mutant line among *GBA1* mutant lines as D409H mutation has a direct impact on the folding of the enzyme, leading to the accumulation of misfolded enzyme in the ER and eventual degradation by the UPS. Furthermore, following MG132 treatment, a significant increase in monomeric α‐synuclein levels was detected in dopaminergic neurons of the PD N370S/WT line, compared to both wild‐type lines and the iPD line. This suggests that in the presence of GBA1 mutations in PD, the degradation of monomeric α‐synuclein is more depended on proteasomal degradation.

In order to test whether *GBA1* mutations lead to oligomerization and/or aggregation of α‐synuclein in iPSC‐derived dopaminergic neurons, we used the A11 antibody, originally raised against prefibrillar amyloid beta (Aβ) oligomers, with a conformation‐specific sensitivity to oligomeric forms (but not monomers and fibrils) of various amyloidogenic proteins, including α‐synuclein (Kayed et al., 2003). Immunocytochemistry results showed that dopaminergic neurons with homozygous *GBA1* mutations had higher accumulation of oligomeric proteins than the wild‐type lines. Interestingly, lines with L444P and D409H homozygous *GBA1* mutations exhibited a greater accumulation of the oligomeric forms than the lines with N370S homozygous *GBA1* mutations (Figure 2c,d). Our results also revealed that dopaminergic neurons of PD patients (both idiopathic and *GBA1* N370S mutant) showed a significant oligomeric protein accumulation compared to the wild‐type cells. However, the PD line harboring the *GBA1* N370S mutation had a significantly higher amount of oligomeric proteins compared to the idiopathic PD line, suggesting an additive effect of *GBA1* mutations on oligomeric protein, most probably α‐synuclein accumulation (Figure 2c,d).

Overall, all *GBA1* homozygous mutant lines and PD lines showed a significant increase in the accumulation of oligomeric protein/peptide levels in iPSC‐derived dopaminergic neurons. The greater accumulation of oligomeric peptides (presumably α‐synuclein) in severe *GBA1* mutant lines might explain the clinical facts that severe *GBA1* mutations are associated with a higher risk of PD, earlier age of onset, and faster disease progression (Cilia et al., 2016; Gan‐Or et al., 2008; Liu et al., 2016).

### 3.3 Release of α‐synuclein in *GBA1* mutant iPSC‐derived dopaminergic neurons

**FIGURE 3:**
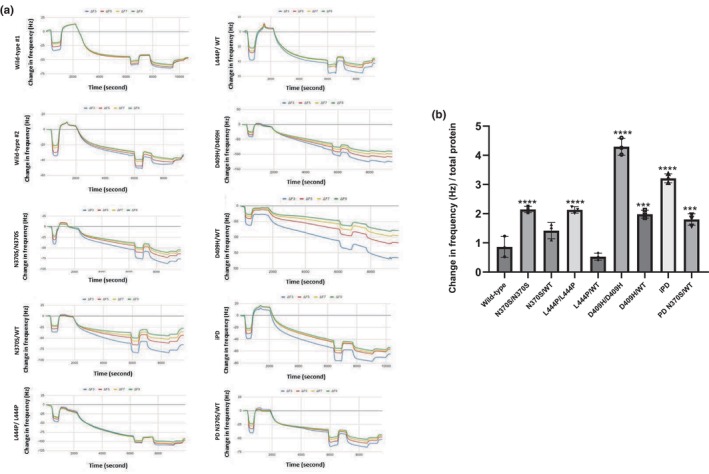
α‐synuclein release in induced pluripotent stem cell (iPSC)‐derived dopaminergic neurons with *GBA1* mutations using quartz crystal microbalance with dissipation (QCM‐D) technique. (a) Frequency change over time graphs obtained from dQCM analysis of culture media collected from differentiated iPSC‐derived dopamine neuron cultures at Day 90. The curves displayed in yellow, blue, green, and red represent measurements at different frequency values for each sample. The quantification of the data utilized the average of values obtained from all frequency ranges (ΔF3, ΔF5, ΔF7, and ΔF9). (b) Frequency change graph obtained from the quantification of dQCM data. Results were normalized to total protein levels. Data are shown as mean + standard deviation. Ordinary one‐way ANOVA, *F*(8, 18) = 87.77, *p* < 0.0001 (*n* = 3 from three different differentiations of iPSC‐derived dopaminergic neurons). Significantly different groups are indicated by *** and **** (***p < 0.001 and ****p < 0.0001).

Related to the pathogenesis of *GBA1*‐associated PD, many studies have shown that mutant GCase might affect the release of α‐synuclein, as well as triggering its accumulation (Bae et al., 2014; Gegg et al., 2020; Migdalska‐Richards et al., 2020). To investigate the release of α‐synuclein, iPSC‐derived dopaminergic neurons were differentiated for 90 days, and the cell culture media was collected and analyzed using the quartz crystal microbalance with dissipation (QCM‐D) technique which successfully allows the detection of the accumulation of aggregation‐prone proteins such as amyloid‐β and α‐synuclein (Buell et al., 2012; Gaspar et al., 2017; Migon et al., 2020). The QCM‐D biosensor surface was coated with recombinant monomeric α‐synuclein monomers and we measured changes in oscillation frequency (Hz) as α‐synuclein molecules in the cell culture media attached to the surface of the biosensor. Our results indicated that *GBA1* mutations led to a significant increase in the release of α‐synuclein into the culture media in GD and PD lines compared to the control lines (Figure 3a,b). The release of α‐synuclein from the dopaminergic neurons was significantly elevated in all homozygous *GBA1* mutant lines. However, this effect was particularly pronounced in the D409H/D409H *GBA1* mutant line (Figure 3a,b). When we compared the heterozygous *GBA1* mutant lines, the release of α‐synuclein from the dopaminergic neurons with D409H/WT genotype was significantly increased. On the other hand, heterozygous N370S/WT and L444P/WT *GBA1* mutant lines did not have a significant increase in the release of α‐synuclein. Overall, the presence of severe homozygous *GBA1* mutations led to a significantly higher amount of oligomeric α‐synuclein accumulation and release, which might explain the clinical severity and relatively rapid progression of PD in severe *GBA1* mutant cases.

### 3.4 Increase in ER stress and ER‐associated degradation (ERAD) in *GBA1* mutant lines

**FIGURE 4:**
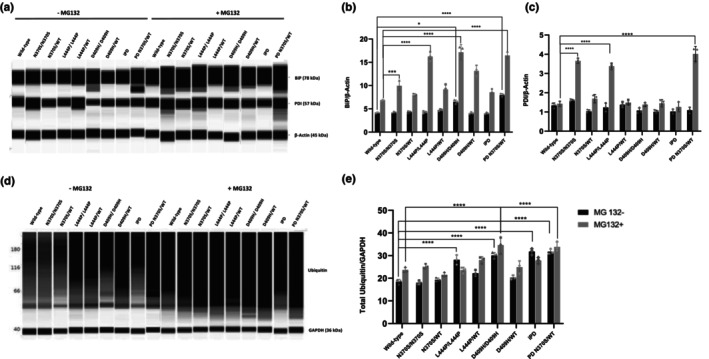
Endoplasmic reticulum (ER) stress and ubiquitin–proteasome system activity in induced pluripotent stem cell (iPSC)‐derived dopaminergic neurons with *GBA1* mutations. (a, d) Immunoblot automated western blot system (WES) analysis of ER stress markers (BiP and PDI) and total ubiquitinated proteins in iPSC‐derived dopamine neuron cultures (Day 40) under basal conditions and after 24‐h treatment with 100 nM MG132 proteasome inhibitor. Graphs depicting the optical densities of (b) BiP, (c) PDI proteins, and (e) total protein ubiquitination bands normalized to β‐Actin or GAPDH expression in wild‐type and *GBA1* mutant cells. Data are shown as mean + standard deviation. (b) Two‐way ANOVA, *F*(8, 18) = 32.77, *p* < 0.0001 (*n* = 3 from three different differentiations of iPSC‐derived dopaminergic neurons), (c) two‐way ANOVA, *F*(8, 18) = 57.43, *p* < 0.0001 (*n* = 3 from three different differentiations of iPSC‐derived dopaminergic neurons), and (e) two‐way ANOVA, *F*(8, 18) = 8.38, *p* < 0.0001 (*n* = 3 from three different differentiations of iPSC‐derived dopaminergic neurons). Significantly different groups are indicated by *, ***, and **** (*p < 0.05, ***p < 0.01, and ****p < 0.0001).

Accumulation of misfolded proteins in the ER induces ER stress, leading to the activation of the unfolded protein response (UPR). In this case, activated ER chaperones correct the folding of misfolded proteins. Misfolded proteins not corrected by chaperones are removed through ERAD in the cytoplasm via UPS (Lu et al., 2010). *GBA1* mutations are known to activate the UPR by causing defects in the folding and stabilization of the GCase enzyme, which is detected and retained in the ER by the quality control machinery (Ron & Horowitz, 2005). In this study, we aimed to investigate the effects of different *GBA1* mutations on ER stress and the UPS. Accordingly, we analyzed ER stress and the total ubiquitination rates of proteins in *GBA1* mutant iPSC‐derived dopaminergic neurons. The results showed that the expression of the ER‐resident chaperone binding immunoglobulin protein (BiP) was significantly up‐regulated in D409H homozygous *GBA1* mutant line and N370S *GBA1* mutant PD line compared with wild‐type iPSC‐derived dopaminergic neurons under basal conditions (Figure 4a,b). When neurons were treated with the proteasome inhibitor MG132, all *GBA1* homozygous lines and the N370S *GBA1* mutant PD line showed increased BiP protein levels compared with wild types (Figure 4a,b). Protein levels of another UPR mediator, protein disulfide isomerase (PDI), did not show significant changes between the cell lines under basal conditions; however, PDI levels were significantly up‐regulated in N370S and L444P homozygous lines and N370S *GBA1* mutant PD lines compared with controls upon MG132 treatment (Figure 4a,c).

The pathology of α‐synuclein accumulation in PD might be related to impairments in the UPS. In previous studies, impairment in UPS and changes in the levels of ubiquitination of neuronal proteins were reported in PD (Deger et al., 2015). In this study, we tested whether *GBA1* mutations affect total ubiquitination levels in iPSC‐derived dopaminergic neurons. The total ubiquitination levels of proteins were calculated by measuring the intensity of bands within the molecular weight range of 40–230 kDa. Our results showed that the amount of total ubiquitinated proteins in cells increased significantly in the cells with homozygous L444P and D409H *GBA1* mutations, idiopathic PD cells, and N370S *GBA1* mutant PD cells (PD N370S/WT) under basal conditions (Figure 4d,e). When neuronal cultures were treated with the proteasome inhibitor MG132, a significant increase in the amount of total ubiquitinated protein was detected only in neurons with the D409H/D409H genotype compared with the control cells (Figure 4d,e).

### 3.5 Impaired chaperone‐mediated autophagy in *GBA1* N370S dopaminergic neurons

**FIGURE 5:**
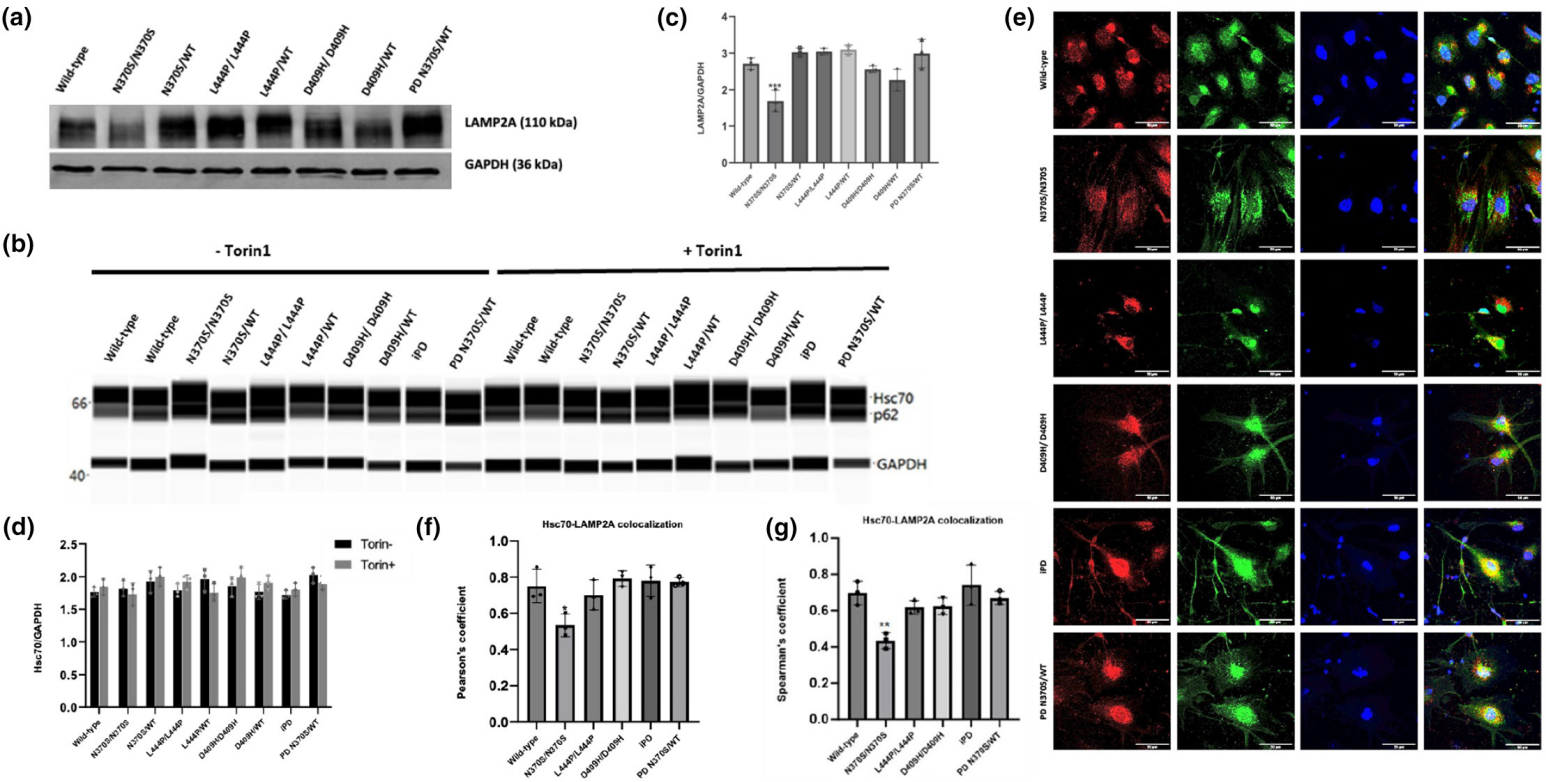
The effect of *GBA1* mutations on chaperone‐mediated autophagy (CMA) in induced pluripotent stem cell (iPSC)‐derived dopaminergic neurons. (a) Western blot analysis of LAMP2A and (b) WES analysis of HSC70 proteins in iPSC‐derived dopamine neuron cultures (Day 40) under basal conditions and after 24‐h treatment with 100 nM MG132 proteasome inhibitor. Graphs depicting the optical densities of (c) LAMP2A and (d) HSC70 bands normalized to GAPDH expression in wild‐type and *GBA1* mutant dopaminergic neurons. Data are shown as mean + standard deviation. (c) Ordinary one‐way ANOVA, *F*(7, 16) = 14.24, *p* < 0.0001 (*n* = 3 from three different differentiations of iPSC‐derived dopaminergic neurons). (d) Two‐way ANOVA, *F*(8, 18) = 1.326, *p* = 0.2927 (*n* = 3 from three different differentiations of iPSC‐derived dopaminergic neurons). (e) Immunofluorescence images of HSC70 (red), LAMP2A (green), and nuclei (blue) in iPSC‐derived dopaminergic neurons (Day40). (f) Quantification of co‐localization between HSC70 and LAMP2a in lysosomes using Pearson's coefficient. Ordinary one‐way ANOVA, *F*(5, 12) = 5.872, *p* = 0.0057 (*n* = 3 from three different differentiations of iPSC‐derived dopaminergic neurons and at least 20 cells per condition). (g) Quantification of co‐localization between HSC70 and LAMP2a in lysosomes using Spearman's rank correlation coefficient. Ordinary one‐way ANOVA, *F*(5, 12) = 8.817, *p* = 0.001 (*n* = 3 from three different differentiations of iPSC‐derived dopaminergic neurons and at least 20 cells per condition). Data are shown as mean + standard deviation. Significantly different groups are indicated by *, **, and *** (*p < 0.05, **p < 0.01, and ***p < 0.001), scale bar: 50 μm.

As α‐synuclein contains a penta‐peptide sequence (_95_VKKDQ_99_) compatible with the KFERQ‐like chaperone‐mediated autophagy recognition motif, it is recognized by the chaperone heat‐shock cognate protein 70 (HSC70) and transported to the lysosome through the lysosomal‐associated membrane protein 2A (LAMP2A) to be degraded in the lysosome (Cuervo et al., 2004). Protein expression levels of LAMP2A were analyzed by immunoblotting in iPSC‐derived dopaminergic neurons (Figure 5a). The results revealed that LAMP2A expression was significantly decreased in N370S *GBA1* homozygous mutant dopaminergic neurons compared to the wild‐type cells (Figure 5c). No significant difference was detected in other lines (Figure 5c). We also analyzed the protein expression levels of HSC70, and the expression of the protein remained unaffected across all cells (Figure 5b,d).

To investigate the interaction of these two proteins, we analyzed the co‐localization of HSC70 and LAMP2A proteins in iPSC‐derived dopaminergic neurons (Figure 5e). Our results showed that HSC70‐LAMP2A co‐localization in N370S *GBA1* homozygous mutant cells was significantly decreased compared to the control lines (Figure 5e,f). Together, these results suggest an impairment in CMA in driven by the N370S *GBA1* mutation that evidently results in dysregulation of α‐synuclein degradation.

### 3.6 Macroautophagy alterations in *GBA1* mutant iPSC‐derived dopaminergic neurons

**FIGURE 6:**
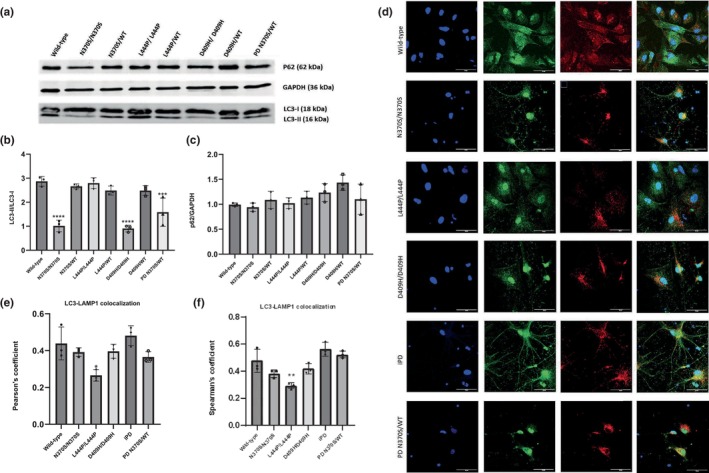
Macroautophagic degradation in induced pluripotent stem cell (iPSC)‐derived dopaminergic neurons with *GBA1* mutations. (a) Western blot analysis of LC3 and p62 proteins in iPSC‐derived dopamine neuron cultures (Day 40). Graphs showing the optical densities of (b) LC3‐II/LC3‐I and (c) p62 bands normalized to GAPDH expression in wild‐type and *GBA1* mutant dopaminergic neurons. Data are shown as mean + standard deviation. (b) Ordinary one‐way ANOVA, *F*(7, 16) = 25.84, *p* < 0.0001 (*n* = 3 from three different differentiations of iPSC‐derived dopaminergic neurons). (c) Ordinary one‐way ANOVA, *F*(7, 16) = 2.746, *p* = 0.0447 (*n* = 3 from three different differentiations of iPSC‐derived dopaminergic neurons). (d) Immunofluorescence images of LAMP1 (red), LC3 (green), and nuclei (blue) in iPSC‐derived dopaminergic neurons (Day40). (e) Quantification of co‐localization between LC3 and LAMP1 in dopaminergic neurons using Pearson's coefficient. Ordinary one‐way ANOVA, *F*(5, 12) = 6.580, *p* = 0.0036 (*n* = 3 from three different differentiations of iPSC‐derived dopaminergic neurons and at least 20 cells per condition). (f) Quantification of co‐localization between LC3 and LAMP1 in dopaminergic neurons using Spearman's rank correlation coefficient. Ordinary one‐way ANOVA, *F*(5, 12) = 13.66, *p* = 0.0001 (*n* = 3 from three different differentiations of iPSC‐derived dopaminergic neurons and at least 20 cells per condition). Data are shown as mean + standard deviation. Significantly different groups are indicated by *, **, *** and **** (*p < 0.05, **p < 0.01, ***p < 0.001, and ****p < 0.0001), scale bar: 50 μm.

To investigate the effects of *GBA1* mutations on the macroautophagic degradation process, we analyzed the expression levels of microtubule‐associated protein light chain 3A (MAP1LC3A or LC3) and p62 protein (Figure 6a). Our results revealed that the LC3‐II/LC3‐I ratio significantly decreased in cells with N370S/N370S, D409H/D409H, and PD N370S/WT genotypes compared to the wild‐type dopaminergic neurons (Figure 6b) suggestive of impaired autophagosome activation in these neurons. However, when comparing p62 protein expression levels between the groups, no significant alterations were identified (Figure 6c).

To examine autophagosome–lysosome fusion, we determined the co‐localization of LC3 and lysosomal‐associated membrane protein 1 (LAMP1) using immunofluorescent staining (Figure 6d). The co‐localization of LC3‐LAMP1 was significantly reduced in L444P/L444P and PD N370S/WT cells compared to the wild‐type lines (Figure 6e). This indicates a possible impairment in autophagosome–lysosome fusion in these cells. Overall, we found that cells with N370S/N370S and D409H/D409H genotypes had defects in the LC3‐II autophagosome formation stage compared to the control cells. On the other hand, in cells with the L444P/L444P genotype, a decrease in autophagosome–lysosome fusion was detected, but no deficits in the autophagosome formation process. The PD line with the N370S/WT *GBA1* mutation showed defects in both autophagosome formation and autophagosome–lysosome fusion.

## 4 DISCUSSION

*GBA1* mutations are well established as the most common genetic risk factor for PD and other synucleinopathies. *GBA1* mutations have varying degrees of impact on both GD and *GBA1*‐associated synucleinopathies. While certain mutations contribute to the development of GD, others (such as E326K and T369M) primarily elevate the risk of Parkinson's disease (Menozzi & Schapira, 2021). *GBA1* mutations differentially contribute to PD risk, impacting disease onset age and the severity of symptoms. However, the precise effects of individual mutations remain poorly understood because of the complicated nature of the underlying molecular mechanisms.

Our study used iPSC‐derived dopaminergic neurons obtained from individuals with various homozygous and heterozygous *GBA1* mutations to elucidate the underlying molecular mechanisms responsible for the observed variations in mutation impact. We generated and characterized iPSC lines obtained from GD patients with N370S/N370S, L444P/L444P, and D409H/D409H genotypes. Additionally, we included obligate heterozygous carriers of *GBA1* gene mutations (with N370S/WT, L444P/WT, and D409H/WT genotypes) derived from these patients' families, a PD patient bearing *GBA1* mutation (N370S/WT), an idiopathic PD patient, and individuals without *GBA1* mutations as healthy controls (Table S1). Our investigation focused on pathways of α‐synuclein accumulation in dopaminergic neurons derived from this panel of *GBA1* mutant iPSCs.

Our findings confirm that homozygous *GBA1* mutations lead to decreased GCase activity and expression, consistent with previous reports (Sanchez‐Martinez et al., 2016; Schondorf et al., 2014). The observed reduction in enzyme activity suggests that *GBA1* mutations disrupt its catalytic function, impacting substrate binding, co‐activation, or folding. Interestingly, the change in enzyme activity was not directly correlated with mutation severity. In contrast to the enzyme activity data, severe mutations (L444P and D409H) exhibited a greater reduction in protein expression compared to the mild mutation (N370S), possibly because of misfolding and ER‐associated degradation (Ron & Horowitz, 2005). When we compared the data from carriers with heterozygous *GBA1* gene mutations, GCase activity was decreased in the N370S/WT and L444P/WT genotypes compared to wild‐type cells. In contrast, the D409H/WT genotype displayed no change in GCase enzyme activity but had a significant decrease in expression. This discrepancy is likely because of the impacts of *GBA1* mutations on the structure of the GCase enzyme. N370S and L444P mutations are believed to influence regions responsible for substrate and co‐activator binding, while the D409H mutation impacts enzyme folding, leading to the accumulation of misfolded enzyme in the ER and eventual degradation (Ron & Horowitz, 2005). These findings align with a previous report, which suggested that certain mutations interfere with substrate and co‐activator binding, while others influence enzyme folding (Vilageliu & Grinberg, 2017).

We hypothesized that disruption of cellular degradation mechanisms such as UPS, CMA, and macroautophagy might differentially contribute to the accumulation of α‐synuclein in iPSC‐derived dopaminergic neurons with different *GBA1* genotypes. Neurons from PD patients with the N370S/WT genotype had increased monomeric α‐synuclein levels compared to both idiopathic PD (iPD) and wild‐type cells. This increase implies that *GBA1* mutations could lead to α‐synuclein accumulation in the context of PD. Expanding our analysis to the accumulation of high‐molecular‐weight oligomeric forms of α‐synuclein, we observed significant accumulation in all homozygous *GBA1* mutant iPSC‐derived dopaminergic neurons (N370S/N370S, L444P/L444P, and D409H/D409H), as well as in dopaminergic neurons from PD patients (both iPD and PD N370S/WT), when compared with WT cells. This accumulation was increased in cells carrying severe homozygous *GBA1* mutations compared to cells with mild homozygous *GBA1* mutations, suggesting that severe homozygous *GBA1* mutations have a greater impact on the accumulation of oligomeric α‐synuclein than mild homozygous *GBA1* mutations. Similarly, previous studies have reported that more accumulation of intracellular α‐synuclein observed in the various cell lines over‐expressing severe mutant *GBA1* compared to the ones over‐expressing mild mutant *GBA1* (Cullen et al., 2011). This observation also agrees with findings from a study by Maor et al., in which the over‐expression of *GBA1* mutants (N370S and L444P) in neuronal cell lines led to a greater accumulation of both monomeric and oligomeric α‐synuclein in the severe L444P mutant compared to the mild N370S mutant (Maor et al., 2019). They reported significant aggregation of α‐synuclein in SHSY5Y cells over‐expressing human L444P GCase but not N370S GCase (Maor et al., 2019). Another study using iPSC‐derived dopamine neurons from PD patients carrying homozygous *GBA1* mutations (N370S/N370S and N370S/c0.84dupG) and GD patients with N370S/N370S and IVS2+1G>T/L444P genotypes showed that the presence of the severe homozygous *GBA1* mutations leads to significant accumulation of α‐synuclein protein compared to cells with homozygous mild *GBA1* mutations in iPSC‐derived dopaminergic neurons (Aflaki et al., 2016). Together, these data indicate that severe *GBA1* mutations cause greater accumulation of α‐synuclein in dopaminergic neurons. In addition to these in vitro studies, a recent study showed that total α‐synuclein levels were significantly higher in CSF samples from PD patients with severe *GBA1* mutations compared to PD patients with mild or no *GBA1* mutations (Lerche et al., 2020).

We also measured α‐synuclein release from iPSC‐derived dopaminergic neurons. We found increased α‐synuclein release from iPSC‐derived dopaminergic neurons across all homozygous *GBA1* mutant lines (N370S/N370S, L444P/L444P, and D409H/D409H) and PD lines (iPD and PD N370S/WT), in comparison with WT cells. Remarkably, in neurons with homozygous *GBA1* mutations, those with L444P and D409H mutations released more α‐synuclein than those with the N370S mutation. These findings suggest that the accumulation and release of oligomeric α‐synuclein is influenced more by severe homozygous *GBA1* mutations. Considering the importance prion‐like mechanisms in PD progression, pathologic α‐synuclein would be expected to transfer more efficiently between neurons in the context of severe *GBA1* mutations.

These findings align with the clinical data presented by Gan‐Or et al., which demonstrated that individuals with severe *GBA1* mutations associated with PD present with a more severe phenotype, characterized by earlier onset of symptoms and more pronounced motor, psychiatric, cognitive, and olfactory deficits (Gan‐Or et al., 2008, 2009). Therefore, it is highly likely that the more severe PD symptoms associated with *GBA1* mutations arise from the strong effect of *GBA1* mutations on the accumulation and release of oligomeric α‐synuclein.

The misfolded GCase enzyme accumulation within the ER caused by *GBA1* mutation is known to trigger an unfolded protein response and proteasomal degradation via ER stress induction. In our model, we observed a significant increase in ER stress and ubiquitination and heightened sensitivity to ER stress in D409H homozygous *GBA1* mutant neurons. These findings are consistent with the observed decrease in GCase expression, which is attributed to the misfolding of GCase associated with the D409H mutation. Furthermore, our findings are in line with previous research that identified disrupted ER homeostasis, elevated markers of ER stress, and activation of the UPR in iPSC‐derived dopaminergic neurons from PD patients, as a consequence of the accumulation of misfolded GCase (Fernandes et al., 2016). The consistency of our data with earlier studies further highlights the effect of *GBA1* mutations on ER‐related processes and pathological mechanisms underlying PD. Only PD neurons with the N370S/WT genotype had elevated ER stress markers under standard culture conditions, and both idiopathic PD (iPD) cells and PD N370S/WT cells exhibited significantly higher levels of total ubiquitination. Upon treatment with a proteasome inhibitor, all homozygous *GBA1* mutant cells, regardless of the specific mutation, had higher ER stress markers which was more pronounced in severe *GBA1* mutations (L444P and D409H). Moreover, the presence of severe homozygous *GBA1* mutations resulted in a significant increase in overall cellular ubiquitination compared to wild‐type cells, aligning with the reduced activity and expression of the mutant GCase in the presence of these mutations. Collectively, these findings provide evidence of the complex relationship between *GBA1* mutations, ER stress, ubiquitination, and their collective impact on cellular processes, providing insights into the disease‐causing mechanisms related to *GBA1* mutations in PD.

Dopaminergic neurons are known to be particularly sensitive to unfolded, misfolded, and excessively accumulative protein inclusions (Michel et al., 2016). In the context of PD pathology, disruptions in ER stress and autophagic degradation pathways contribute to dopaminergic neuron loss and cellular proteostasis imbalance, driven by chaperone‐mediated autophagy (CMA) and macroautophagy. Our study revealed significant reduction in the expression of the CMA‐specific lysosomal membrane protein LAMP2A and its co‐localization with HSC70 in iPSC‐derived dopaminergic neurons with N370S/N370S genotype using Parson's and Spearman's correlations. This might suggest that dysfunctional CMA is restricted to cases with mild homozygous *GBA1* mutations. In post‐mortem brain tissues of PD patients, decreased levels of HSC70 and LAMP2A were reported, indicating a decrease in CMA (Alvarez‐Erviti et al., 2010; Murphy et al., 2015).

Similar to CMA, macroautophagy is also differentially affected by different *GBA1* mutations in iPSC‐derived dopaminergic neurons. Lipidation of LC3, which is involved in autophagosome formation and degradation of the insoluble fibrillar form of α‐synuclein, was significantly decreased in dopaminergic neurons with N370S/N370S and D409H/D409H genotypes compared to wild‐type cells. Furthermore, co‐localization analysis of LC3 and the lysosomal marker LAMP1 indicated a significant reduction in co‐localization in cells with the L444P/L444P genotype compared to wild‐type cells. The decreased LC3‐LAMP1 co‐localization suggests that autophagosome–lysosome fusion is disrupted. Disruption in the macroautophagic flux was correlated with oligomeric α‐synuclein accumulation, consistent with the fact that macroautophagy is thought to target the oligomeric and/or aggregated form of α‐synuclein for degradation (Lee et al., 2004). Existing literature also supports the possibility that accumulated α‐synuclein in cells can disrupt various stages of the macroautophagy mechanism, such as vesicular trafficking (Winslow et al., 2010), cellular cytoskeleton (Sarkar et al., 2021), and autophagosome–lysosome fusion (Stykel et al., 2021).

These co‐localizations were found to be significant using both of Pearson's and Spearman's correlation coefficients. Although these two methods are widely accepted for the co‐localization analysis of autophagic markers (Orvedahl et al., 2011; Tumbarello et al., 2012; Zhang et al., 2019), there are distinct differences between two methods. While Pearson's correlation measures the strength and direction of the linear relationship between two continuous variables, assuming normality and linearity, Spearman correlation assesses the monotonic relationship between variables, making it suitable for ordinal, interval, or ratio data, and it is more robust to outliers (Adler et al., 2008). This adds further confidence to our findings.

PD cells with the N370S/WT genotype exhibited disruptioned autophagosome–lysosome fusion. According to these findings, disruptions in the macroautophagic flux in N370S/WT PD cells, where monomeric and oligomeric α‐synuclein accumulation is higher, are consistent with existing literature (Woodard et al., 2014). The increased cellular accumulation of α‐synuclein caused by the presence of *GBA1* mutation (N370S/WT) leads to errors in vesicular trafficking, resulting in disruptions in the macroautophagy mechanism. In this context, it can be suggested that the accumulation of α‐synuclein caused by *GBA1* mutations synergistically contributes to PD pathology by affecting cellular degradation mechanisms.

A limitation of using iPSC‐derived dopaminergic neurons from this genetically diverse cohort is the wide age range of donors, spanning from 10 to 49 years (see Table S1). This variability in age could potentially introduce variations in reprogramming efficiency, differentiation capacity, and the presence of age‐related phenotypes. However, studies suggest that the process of reprogramming primary fibroblast cells derived from biopsy samples, achieved through the introduction of Yamanaka factors, effectively resets these cells to an embryonic state, mitigating the expression of age‐associated traits commonly observed in older cells (Strassler et al., 2018). Several studies indicate that reprogrammed iPSCs from both young and old individuals have similar reprogramming efficiency, differentiation potential, and markers associated with aging (Chang et al., 2011). Furthermore, even the aging factors seen in iPSCs obtained from donors of varying ages have been observed to revert to a more embryonic‐like state (Prigione et al., 2011). Altogether, these data suggest that the age of the donor is unlikely to significantly influence the establishment of iPSC‐derived dopaminergic neuron models.

iPSC‐derived disease models allow investigation of mechanisms of neurodegeneration, while overcoming challenges such as restricted access to affected neurons, limited availability of post‐mortem tissue, and ethical concerns. However, a limitation of iPSC‐derived disease models is the use of a limited number of patient‐derived lines in studies. This limitation, along with challenges such as genetic heterogeneity and variable phenotypes, requires acknowledgment for accurate research interpretation. Collaborative efforts, larger sample sizes, standardized protocols, and advanced analytical methods will help to address these limitations and enhance the reliability and robustness of iPSC‐based studies in the future.

**FIGURE 7:**
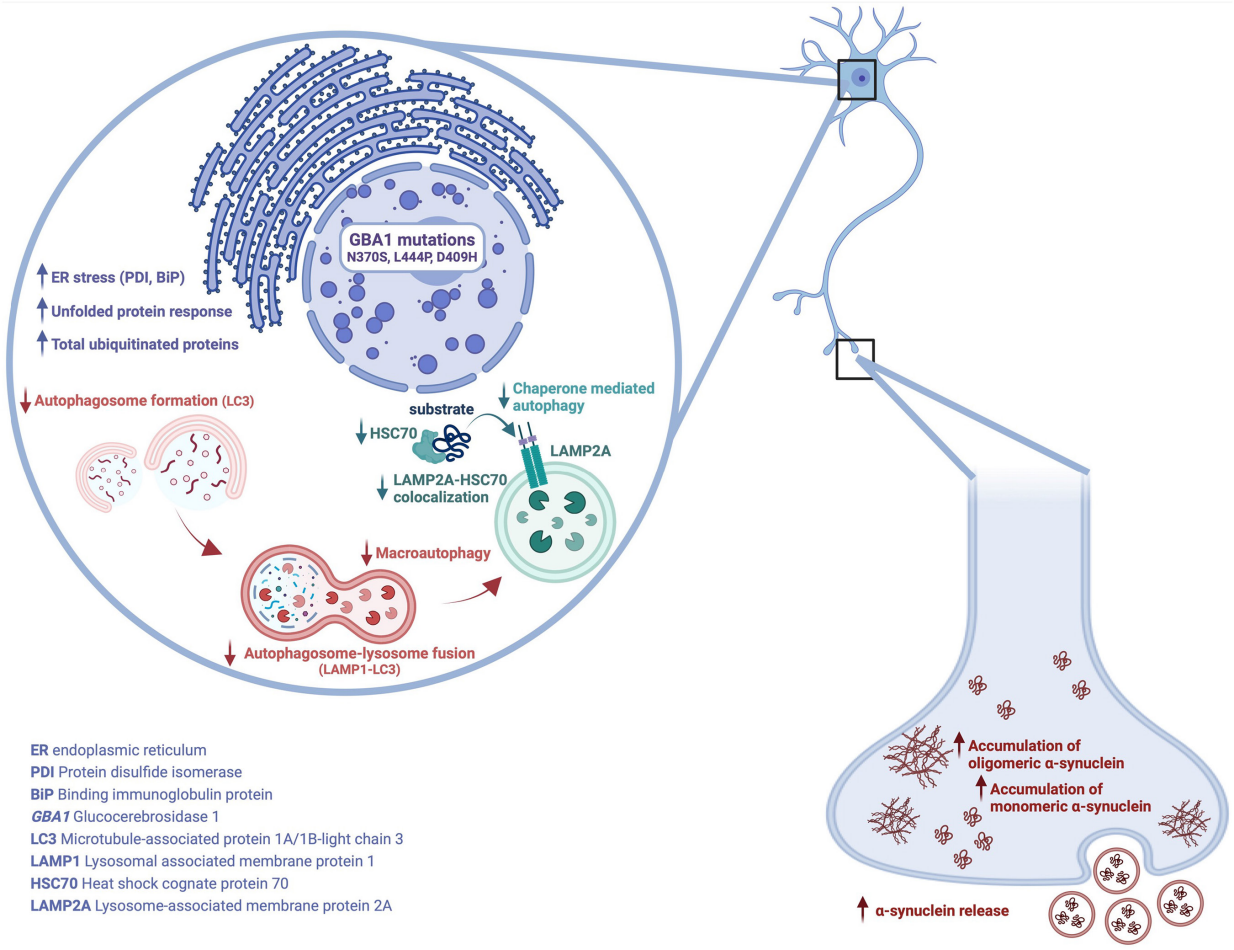
A summary of the effects of *GBA1* mutations on patient‐derived dopaminergic neurons. Impairments in ubiquitin proteasome system, chaperone‐mediated autophagy, and macroautophagy mechanisms in the presence of *GBA1* mutations lead the accumulation monomeric and oligomeric forms of α‐synuclein and increase the release of α‐synuclein.

In conclusion, we show that *GBA1* mutations induce α‐synuclein accumulation in iPSC‐derived dopaminergic neurons because of proteasomal/lysosomal dysfunction. Although both gain‐of‐function and loss‐of‐function hypotheses have been proposed to explain *GBA1*‐dependent α‐synuclein accumulation, our findings lend support to the gain‐of‐function hypothesis wherein mutant GCase contributes to ER stress and autophagic impairment, promoting monomeric/oligomeric α‐synuclein accumulation (Figure 7). Moreover, the study highlights the importance of comparing the molecular effects of severe and mild *GBA1* mutations to understand their differential impact on phenotype and the progression of PD‐associated neurodegeneration. Understanding these mechanisms may aid in the development of targeted therapeutic strategies and personalized treatment approaches for individuals with *GBA1*‐associated disorders. This knowledge might aid in identifying individual risk factors and potential biomarkers for *GBA1*‐related PD and associated synucleinopathies.

## AUTHOR CONTRIBUTIONS

**G. Onal:** Investigation; validation; project administration; writing – review and editing; writing – original draft; methodology; visualization; formal analysis; funding acquisition. **G. Yalçın‐Çakmaklı:** Investigation; methodology; formal analysis; writing – review and editing; visualization. **C. E. Özçelik:** Methodology; validation; writing – review and editing; investigation. **I. Boussaad:** Methodology; investigation; validation; formal analysis; software; data curation; supervision; writing – review and editing. **U. Ö. Ş. Şeker:** Writing – review and editing; formal analysis; methodology; resources. **Hugo J. R. Fernandes:** Writing – review and editing; supervision. **H. Demir:** Resources; writing – review and editing. **R. Krüger:** Resources; supervision; writing – review and editing; conceptualization; methodology. **B. Elibol:** Resources; writing – review and editing; supervision; conceptualization. **S. Dökmeci:** Conceptualization; supervision; resources; funding acquisition; writing – review and editing; validation; project administration; investigation; writing – original draft; methodology. **M. M. Salman:** Conceptualization; funding acquisition; writing – original draft; writing – review and editing; supervision; formal analysis; investigation; validation; methodology.

## CONFLICT OF INTEREST STATEMENT

We report no conflicts of interest.

## Supporting information


Table S1.
Figure S1.
Figure S2.
Figure S3.
Figure S4.

## Data Availability

The data that support the findings of this study are available from the corresponding author upon reasonable request.
