# Supplementary material for: Variant‐specific effects of GBA1 mutations on dopaminergic neuron proteostasis
Source: J Neurochem. 2024 Apr 20;168(9):2543–60. doi: 10.1111/jnc.16114 (PMC11898552; doi:10.1111/jnc.16114)
Supplement: Supplementary file 1 — Table S1. Figure S1. Figure S2. Figure S3. Figure S4. [file JNC-168-2543-s001.pdf]

VARIANT SPECIFIC EFFECTS OF *GBA1* MUTATIONS ON DOPAMINERGIC NEURON PROTEOSTASIS

Onal, G.

University of Oxford, Department of Physiology, Anatomy and Genetics, Kavli Institute for NanoScience Discovery, Oxford, UK

University of Oxford, Oxford Parkinson's Disease Centre, Oxford, UK

Yalçın-Çakmaklı, G.

Hacettepe University, Faculty of Medicine, Department of Neurology, Ankara, Turkey.

Özçelik, C. E.

Bilkent University, UNAM-Institute of Materials Science and Nanotechnology, National Nanotechnology Research Center, Ankara, Turkey

Boussaad, I.

University of Luxembourg, Translational Neuroscience, Luxembourg Centre for Systems Biomedicine (LCSB), Luxembourg

Şeker, U. Ö. Ş.

Bilkent University, UNAM-Institute of Materials Science and Nanotechnology, Interdisciplinary Neuroscience Program, National Nanotechnology Research Center, Ankara, Turkey

Fernandes, H. R.

University of Oxford, Department of Physiology, Anatomy and Genetics, Kavli Institute for NanoScience Discovery, Oxford, UK

University of Oxford, Oxford Parkinson's Disease Centre, Oxford, UK

Demir, H.

Hacettepe University, Faculty of Medicine, Departments of Pediatric Gastroenterology, Hepatology and Nutrition, Ankara, Turkey

Krüger, R.

University of Luxembourg, Translational Neuroscience, Luxembourg Centre for Systems Biomedicine (LCSB), Luxembourg

Transversal Translational Medicine, Luxembourg Institute of Health (LIH), Luxembourg

Parkinson Research Clinic, Centre Hospitalier de Luxembourg (CHL), Luxembourg

Elibol, B.

Hacettepe University, Faculty of Medicine, Department of Neurology, Ankara, Turkey

Dökmeci, S.*

Hacettepe University, Faculty of Medicine, Department of Medical Biology, Ankara, Turkey

Salman, M.*

University of Oxford, Department of Physiology, Anatomy and Genetics, Kavli Institute for NanoScience Discovery, Oxford, UK

University of Oxford, Oxford Parkinson's Disease Centre, Oxford, UK

***co-corresponding authors**

Supplementary Table1

Diagnosis	GBA1 Genotype	Gender	Age of biopsy sampling (years)	Clinical symptoms
GD-I	N370S/N370S	F	15	Organomegaly, bilateral Erlenmeyer deformity, storage cells in the liver, short stature, constipation
GBA1 carrier	N370S/WT	F	34	-
GD-III	L444P/L444P	M	10	Splenomegaly, anemia, Erlenmeyer deformity, osteoporosis, epilepsy, neurological involvement, and external gaze paralysis
GBA1 carrier	L444P/WT	M	31	-
GD-III	D409H/D409H	M	12	Splenomegaly, femoral fibrous cortical defect, neurological involvement, epilepsy, apraxia, gaze palsy, and decreased systolic function
GBA1 carrier	D409H/WT	M	35	-
Idiopathic PD	WT/WT	F	49	PD onset age: 44 (5 years), MDS UPDRS III score: 11, LEDD (Levodopa Equivalent Daily Dose): 1396 mg, resting tremor in the left hand
PD	N370S/WT	F	47	PD onset age: 46 (1 year), MDS UPDRS III score: 6, LEDD (Levodopa Equivalent Daily Dose): 525 mg, resting tremor in the left hand, pain and slowness in the left leg, rigidity in the left hand and left leg
Wildtype-1	WT/WT	M	45	-
Wildtype-2	WT/WT	F	42	-

Supplementary Figure 1

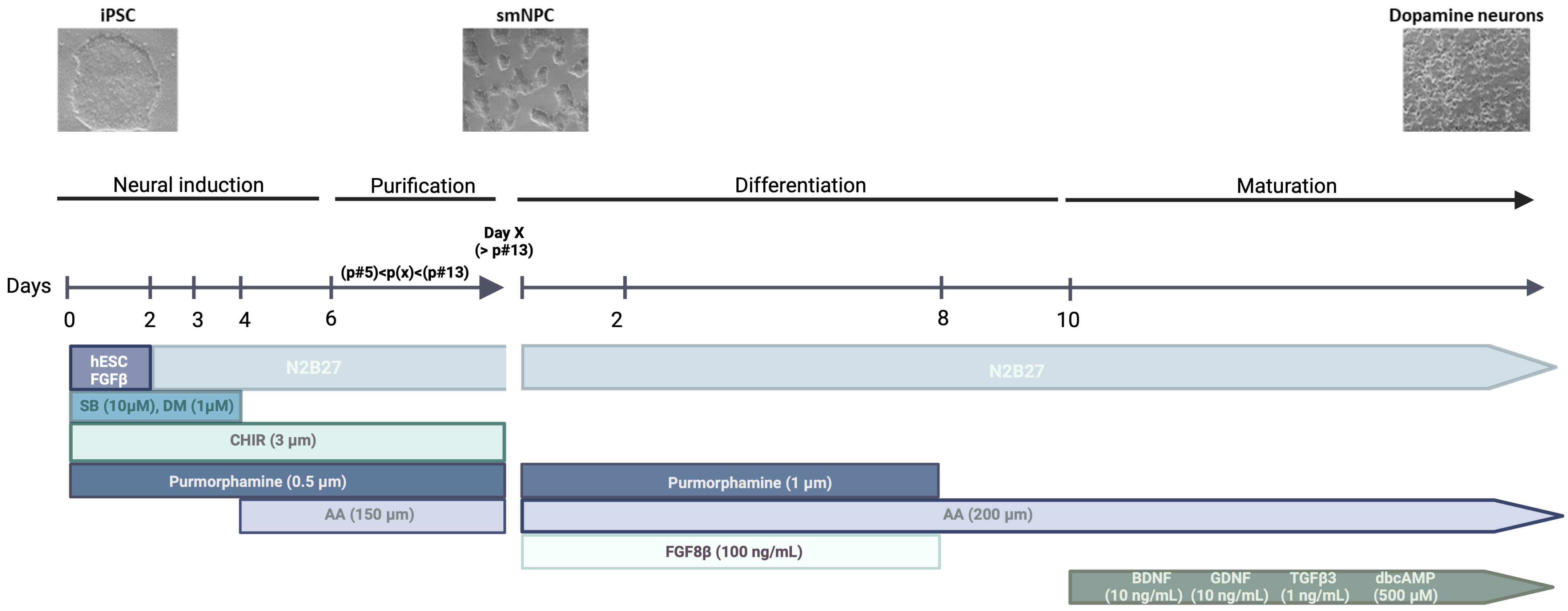

Supplementary Figure 2

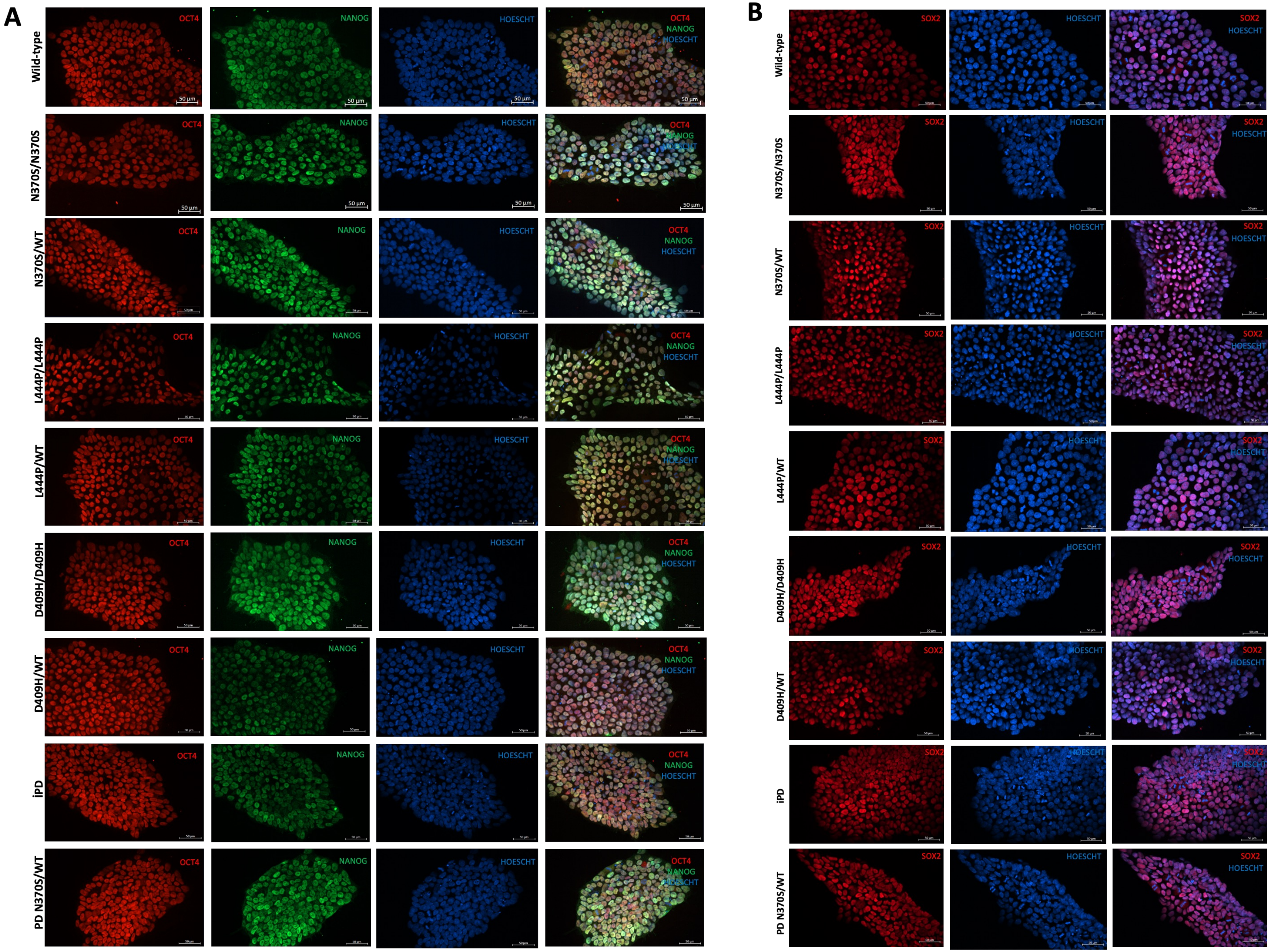

Supplementary Figure 3

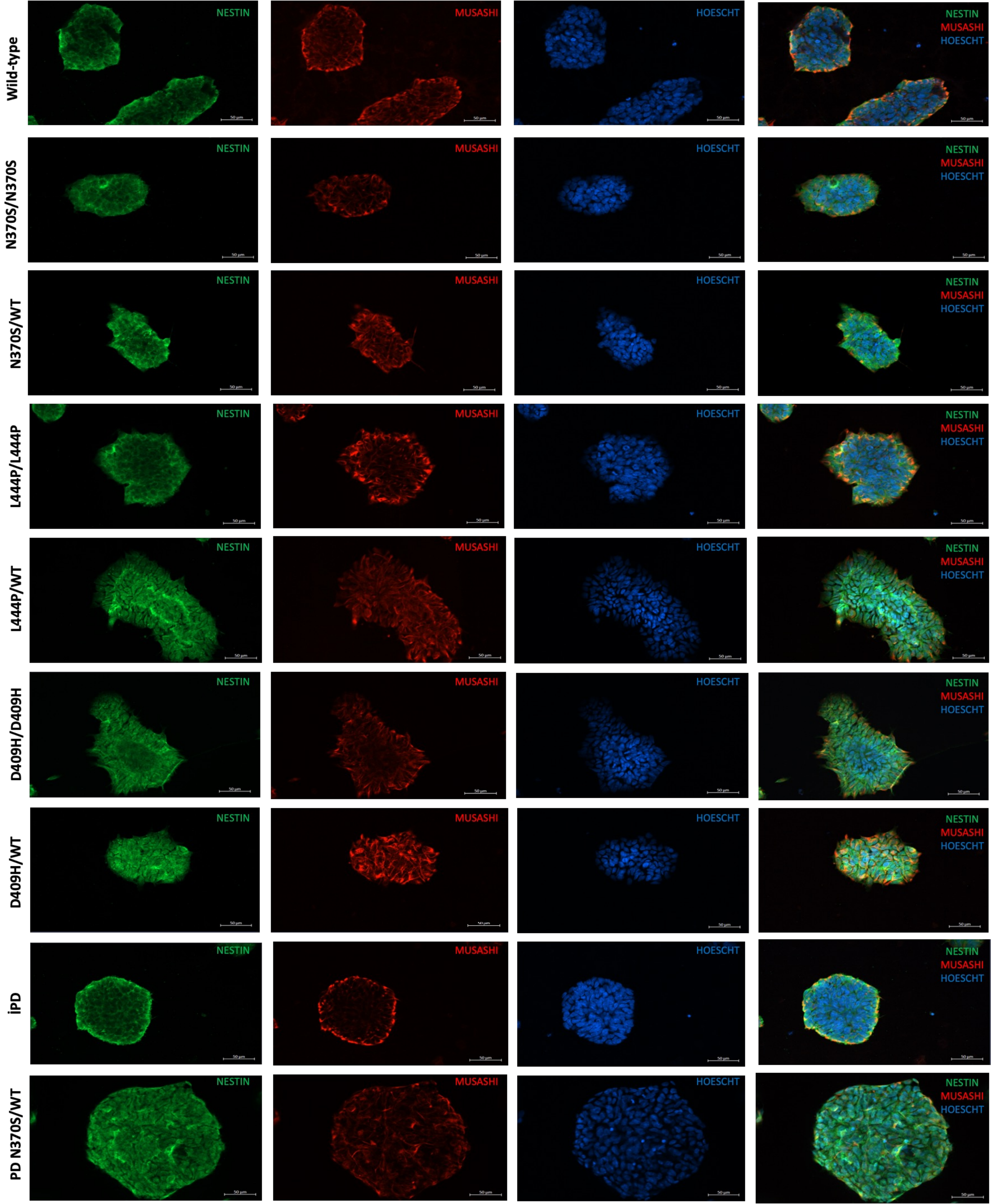

Supplementary Figure 4

A

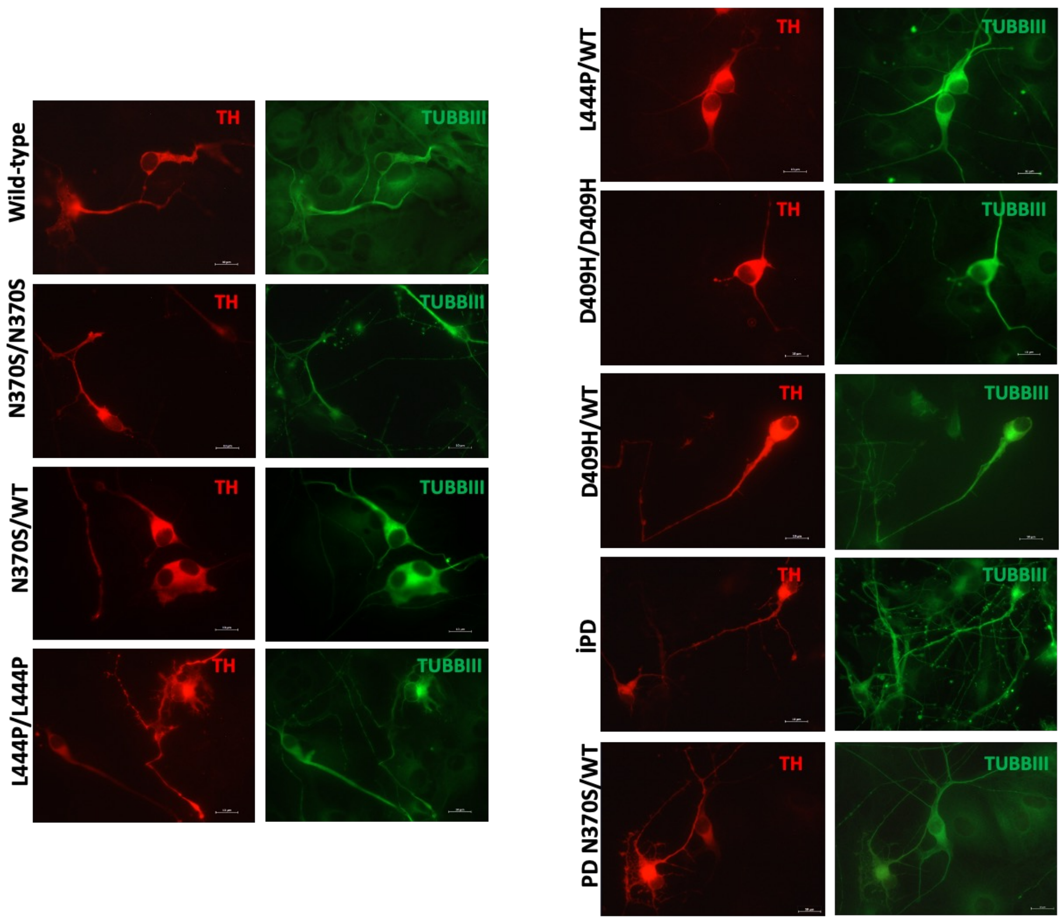

B

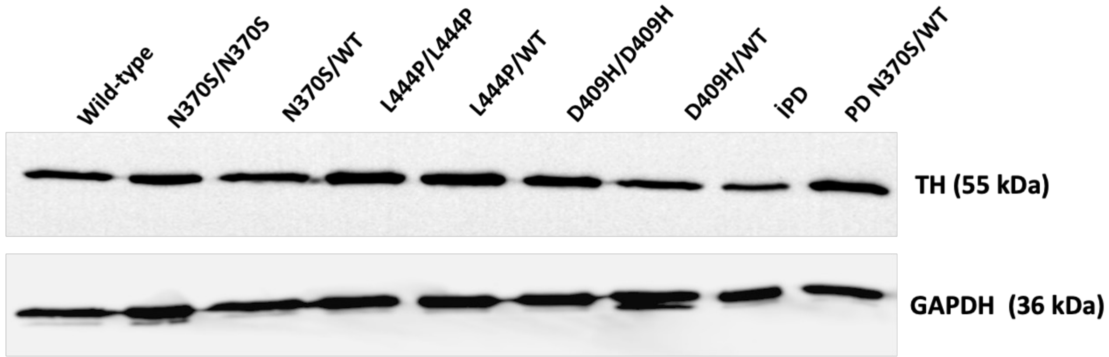

Supplementary Table 1. The demographic information of individuals from whom skin biopsy samples were obtained

Supplementary Figure 1. The differentiation of iPSCs into small molecule neuronal progenitor cells (smNPC) and mature dopaminergic neurons is outlined, following the protocol from Reinhardt et al. (2013). iPSCs were cultured on Matrigel-coated plates in E8 media supplemented with 10 µM SB43152 (SB), 1 µM dorsomorphin (DM), 3 µM CHIR99021 (CHIR), and 0.5 µM purmorphamine for 2 days. Then, the media was changed to N2B27 neuronal cell culture medium composed of 49% Neurobasal medium, 49% DMEM/F12, 1% Glutamax, 1% Penicillin-Streptomycin, 1:100 B-27 Supplement, 1:200 N-2 supplement, with the addition of 150 µM ascorbic acid. smNPCs were purified until a minimum of passage 13. For the initiation of dopaminergic neuron differentiation, smNPCs (>p13) were cultured in N2B27 neuronal media supplemented with 200 µM ascorbic acid, 500 µM db-cAMP, 20 ng/ml BDNF, 10 ng/ml GDNF, and 1 ng/ml TGF-β-III until the specified time point.

Supplementary Figure 2. Immunofluorescence staining images of induced pluripotent stem cell (iPSC) colonies for pluripotency markers (A) OCT4 (red), NANOG (green) and (B) SOX2 (red). Nuclei are stained with Hoescht (blue). Scale bar: 50 µm.

Supplementary Figure 3. Immunofluorescence staining images of small molecule neuronal progenitor cells (smNPCs) for neuronal progenitor markers NESTIN (green) and MUSASHI (red). Nuclei are stained with Hoescht (blue). Scale bar: 50 µm.

Supplementary Figure 4. (A) Immunofluorescence staining images of induced pluripotent stem cell (iPSC) derived dopaminergic neurons for (A) Tyrosine hydroxylase (TH) (red) and Tubulin β-III (green). Scale bar: 10 µm. (B) Western blot analysis of TH and GAPDH proteins in iPSC-derived dopamine neuron cultures (Day 40).
